# Serum glycoprotein non-metastatic melanoma protein B (GPNMB) level as a potential biomarker for diabetes mellitus-related cataract: A cross-sectional study

**DOI:** 10.3389/fendo.2023.1110337

**Published:** 2023-02-16

**Authors:** Da Huo, Yuan-Yuan Liu, Chi Zhang, Lv-Tao Zeng, Guo-Qing Fan, Li-Qun Zhang, Jing Pang, Yao Wang, Tao Shen, Xue-Fei Li, Chuan-Bao Li, Tie-Mei Zhang, Jian-Ping Cai, Ju Cui

**Affiliations:** ^1^ The Key Laboratory of Geriatrics, Beijing Institute of Geriatrics, Institute of Geriatric Medicine, Chinese Academy of Medical Science, Beijing Hospital/National Center of Gerontology of National Health Commission, Beijing, China; ^2^ Shenzhen Institute of Synthetic Biology, Shenzhen Institute of Advanced Technology, Chinese Academy of Sciences, Shenzhen, China; ^3^ Department of Laboratory Medicine, Beijing Hospital, National Center of Gerontology, National Health Commission, Institute of Geriatric Medicine, Chinese Academy of Medical Sciences, Beijing, China

**Keywords:** diabetes mellitus, cataract, GPNMB, biomarker, serum

## Abstract

**Background:**

Diabetes mellitus (DM), a metabolic disease that has attracted significant research and clinical attention over the years, can affect the eye structure and induce cataract in patients diagnosed with DM. Recent studies have indicated the relationship between glycoprotein non-metastatic melanoma protein B (GPNMB) and DM and DM-related renal dysfunction. However, the role of circulating GPNMB in DM-associated cataract is still unknown. In this study, we explored the potential of serum GPNMB as a biomarker for DM and DM-associated cataract.

**Methods:**

A total of 406 subjects were enrolled, including 60 and 346 subjects with and without DM, respectively. The presence of cataract was evaluated and serum GPNMB levels were measured using a commercial enzyme-linked immunosorbent assay kit.

**Results:**

Serum GPNMB levels were higher in diabetic individuals and subjects with cataract than in those without DM or cataract. Subjects in the highest GPNMB tertile group were more likely to have metabolic disorder, cataract, and DM. Analysis performed in subjects with DM elucidated the correlation between serum GPNMB levels and cataract. Receiver operating characteristic (ROC) curve analysis also indicated that GPNMB could be used to diagnose DM and cataract. Multivariable logistic regression analysis illustrated that GPNMB levels were independently associated with DM and cataract. DM was also found to be an independent risk factor for cataract. Further surveys revealed the combination of serum GPNMB levels and presence of DM was associated with a more precise identification of cataract than either factor alone.

**Conclusions:**

Increased circulating GPNMB levels are associated with DM and cataract and can be used as a biomarker of DM-associated cataract.

## Introduction

Diabetes mellitus (DM) is a metabolic disease characterized by high blood glucose levels. Owing to the rapidly increasing numbers of diabetic patients worldwide, it has become one of the most common and insidious chronic diseases with an estimated 4.2 million deaths among 20–79-year-old adults in 2019 (1, 2). In the Middle East and North Africa region, 16.2% of all-cause deaths are attributable to DM. In the South-East Asia and Western Pacific regions, the number of DM-related deaths in 2019 was 1,150,344 and 1,265,051, respectively (2). In addition, another study in 2021 showed that almost a half of all 20–79-year-old adults with DM were unaware of their diabetic status (3). Thus, advances in the study of biomarkers and mechanism are urgently needed for the prediction and therapy for DM.

The progression of DM are often accompanied by several complications such as acute kidney injury, cardiovascular diseases, heart failure, muscle infarction, and cognitive dysfunction (4–8). DM also affects ocular structures, contributes to the pathogenesis of cataract, and causes visual impairment in diabetic patients (9). According to the World Health Organization, cataract is an opacity of the lens with corrected visual acuity less than 0.7. Reports have regarded cataract as the leading cause of blindness globally and indicate that most cases of blindness cases from cataract occur in low- and middle-income countries (10–12). Globally, cost utility values for intraocular lens (IOL) implant surgery, one of the cost-effective treatments for cataract, remain considerable and cause a heavy public health burden (13). However, except for ocular examination, effective molecular biomarkers from blood indices are rarely discovered for the detection of cataract.

Glycoprotein non-metastatic melanoma protein B (GPNMB), also known as osteoactivin given its role in osteopetrosis in rats (14), is widely expressed in various types of cells such as macrophages (15), dendritic cells (16), osteoblasts (17), melanocytes (18), neurons (19) and hepatocytes (20). GPNMB contains an integrin-binding domain and extracellular heparin which contributes to binding to several types of cells such as vascular endothelial cells, keratinocytes, melanoma cells, fibroblasts and T cells (21–24). Study has identified the role of liver-secreted GPNMB in exacerbating obesity and insulin resistance by promoting lipogenesis (20). Cao C et al.’s (25) research has revealed the correlation of circulating levels of GPNMB with gestational DM. Moreover, the expression level of GPNMB is reportedly associated with type 1 DM-related renal function decline (26). Nevertheless, little is known about the role of GPNMB in diabetic cataract.

We investigated the role of GPNMB as a potential biomarker for DM-related cataract based on the data and samples collected from an ongoing cohort study—China Aging Longitudinal Study (CALS)—that enrolled a total of 26,000 healthy Chinese residents from seven geographic areas with the aim to investigate health and aging trends in China. The serum concentrations of GPNMB were measured, and some indicators of physical examination were analyzed.

## Materials and methods

### Study population

Subjects aged ≥25 years and without psychiatric disorders and alcohol or drug abuse were enrolled in CALS. From this existing cohort, we enrolled subjects from Long Tanhu Community in North China (426 participants) and excluded the following: subjects who required acute medical treatment or hospitalization within the first 3 months of GPNMB measurement (3 participants); those with severe diseases including cardiac, hepatic, or renal disease, and respiratory failure (10 participants); those unable to walk independently (2 participants); and those previously diagnosed with dementia (2 participants) and cancers (3 participants).

Finally, a total of 406 participants (155 men and 251 women; with DM=60, without DM=346) were included in this study. All subjects signed the informed consent form. Among these, 21.43% participants were diagnosed with cataract.

### Clinical and biochemical measurements

Participants who had an opacity in the lens or were previously diagnosed with cataract were defined as the cataract group. Diabetes mellitus was defined by fasting serum glucose ≥126 mg/dL and/or glycosylated hemoglobin (HbA1c) ≥6.5% or those requiring treatment with anti-diabetes medication. All subjects were required to fast for 8 h before screening and filled in questionnaires regarding medical history. Clinical characteristics such as height, weight, body mass index (BMI), and total body fat mass were obtained. Total body fat mass was detected with the body composition analyzer (TsingHua Tong Fang, BCA-2A). Body fat percentage (Fat%) was defined as total body fat mass divided by body weight. Fat mass index (FMI) was defined as total body fat mass divided by the height squared.

Concentrations of fasting blood glucose (GLU), total cholesterol (TC), total triglyceride (TG), high density lipoprotein-C (HDL-C), and low density lipoprotein-C (LDL-C) were assayed using enzymatic methods and detected by Hitachi Automatic Analyzer ((LABOSPECT 008 AS, Japan). Concentrations of insulin and folic acid (FOL) were assayed using a solid-phase enzyme-linked chemiluminescent immunoassay and detected by IMMULITE2000 Automatic Immune Analyzer (Siemens Healthcare Diagnostics, Inc.). HbA1c was measured by nitroblue tetrazolium method and detected by AU680 Automated Biochemical Instrument (Beckman Coulter, Inc.). Insulin resistance status was assessed using the homeostasis model assessment of insulin resistance (HOMA-IR) according to the following formula: fasting serum insulin (μU/mL) × fasting serum glucose (mmol/L)/22.5 (27, 28).

### Serum samples preparation and measurement

After 8 h of overnight fasting, blood samples were obtained *via* venipuncture from the median cubital vein, left in vacutainer tubes with coagulant at room temperature to clot for 15-30 min, centrifuged to collect the supernatant, and finally stored at -80°C until further analysis. Serum GPNMB concentrations were determined with an enzyme-linked immunosorbent assay kit (ELH-Osteoactivin, RayBiotech, Inc, Norcross, GA, USA). The assay had a sensitivity of 45 ng/mL to human GPNMB. The intra-assay and inter-assay coefficients of variation were <10% and <12%, respectively. The assay detection range was 49.15–12000 pg/mL. Serum samples were diluted 10 times before detection and measured according to the manufacturer’s instructions.

### Statistical analysis

Statistical analysis was performed using IBM SPSS Statistics for Mac (version 26.0; IBM Corporation, Armonk, NY, USA) and R script 4.1.0. Continuous variables were presented as mean ± SD or median (interquartile range), while categorical variables were expressed as percentages. Differences between the two groups were analyzed by Wilcoxon rank sum test or Student’s *t*-test. Comparison among three or more groups was performed using one-way analysis of variance (ANOVA) or Kruskal–Wallis H test. Categorical variables were compared using chi-squared test or Fisher’s exact test. The association of study variables with DM or cataract was analyzed by univariable and multivariable logistic regression. The area under the receiver-operating characteristic (ROC) curve was calculated to test its predictive discrimination of DM and cataract. The optimal cut-off value was determined using the maximum sum of sensitivity and specificity based on the Youden index. Two-sided values of P<0.05 were considered to indicate statistically significant differences.

## Results

**Figure 1:**
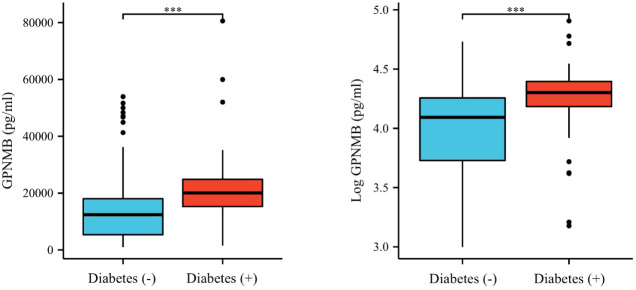
Plasma GPNMB levels depending on the existence of diabetes. ***P < 0.001 using a Wilcoxon rank sum test.

**Figure 2:**
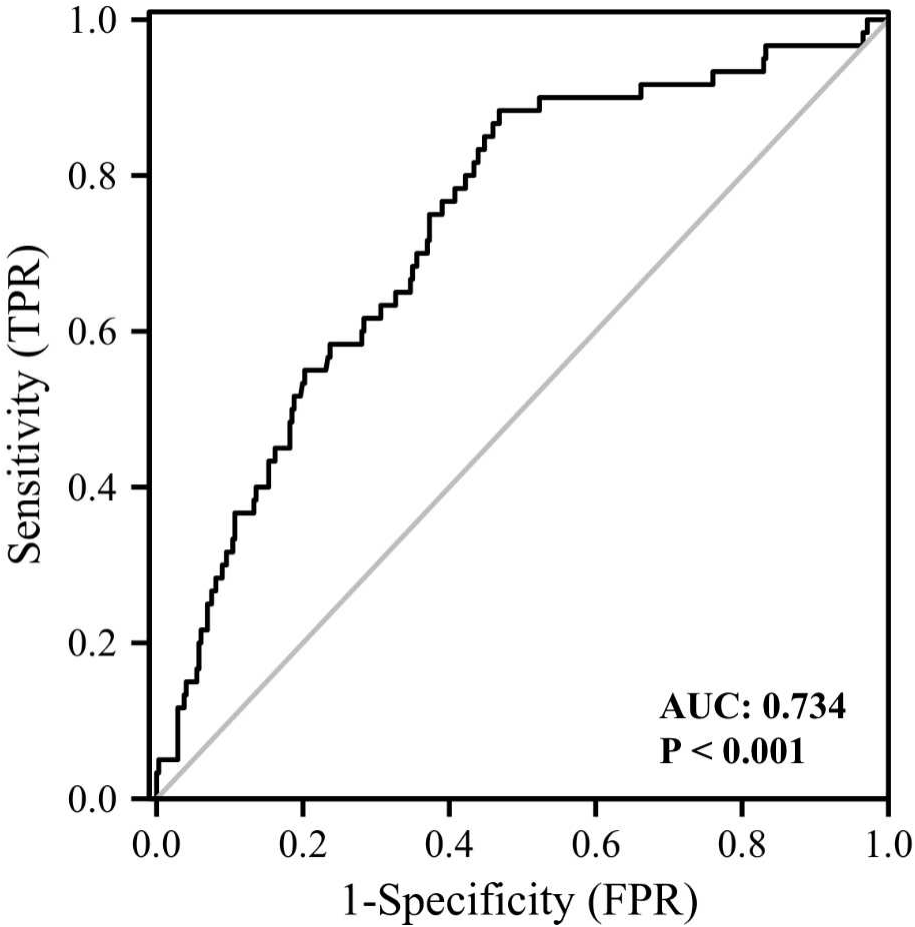
ROC curve analysis of the ability of plasma GPNMB to predict the presence of diabetes. AUC, area under curve.

The clinical and biochemical characteristics of subjects with or without DM are shown in **Table 1**. Subjects with DM were older and had higher BMI, FMI, Fat%, HOMA-IR, HbA1c, TG, FOL, INS, blood glucose and serum GPNMB levels than those without DM. Further, the DM group had a higher proportion of subjects diagnosed with cataract than the non-DM group. HDL-C levels were also lower in the DM than non-DM group. Circulating serum GPNMB levels were higher in subjects with DM than those without DM (**Figure 1**). We further plotted ROC curves based on GPNMB levels which showed a predictive ability of 0.734 to identify DM (asymptotic significance: <0.001) (**Figure 2**). The optimal cut-off value of GPNMB was 12,820.057 pg/mL (88.3% sensitivity and 53.2% specificity) to detect DM.

**Table 1:** Clinical and biochemical characteristics of study participants classified according to diabetes.

Characteristic	Overall	Diabetes (-)	Diabetes (+)	*P* value
Age (years), median (IQR)	50.000 (34.000, 63.750)	48.000 (33.000, 58.000)	66.500 (58.750, 76.000)	< 0.001
Male (%)	38.200	36.990	45.000	0.301
BMI (kg/m^2)^, median (IQR)	23.700 (21.600, 26.040)	23.450 (21.500, 25.830)	24.690 (23.000, 27.230)	0.012
FMI (kg/m^2^), median (IQR)	6.090 (5.038, 7.570)	5.980 (4.880, 7.370)	7.170 (5.920, 8.040)	< 0.001
Fat (%), mean ± SD	26.250 ± 8.2	26.270 ± 5.880	29.360 ± 5.820	0.001
HOMA-IR, median (IQR)	1.720 (1.200, 2.731)	1.640 (1.160, 2.410)	3.050 (1.790, 4.460)	< 0.001
HbA1c (%), median (IQR)	5.800 (5.600, 6.200)	5.800 (5.500, 6.000)	6.400 (5.820, 7.070)	< 0.001
GLU (mmol/l), median (IQR)	5.200 (5.000, 5.700)	5.200 (4.900, 5.600)	6.300 (5.570, 7.080)	< 0.001
TC (mmol/l), median (IQR)	4.740 (4.180, 5.363)	4.750 (4.200, 5.380)	4.620 (4.000, 5.170)	0.123
TG (mmol/l), median (IQR)	0.960 (0.640, 1.470)	0.920 (0.630, 1.400)	1.160 (0.890, 1.630)	0.007
HDL-C (mmol/l), median (IQR)	1.420 (1.190, 1.690)	1.450 (1.230, 1.710)	1.230 (1.060, 1.440)	< 0.001
LDL-C (mmol/l), median (IQR)	2.860 (2.335, 3.502)	2.860 (2.330, 3.510)	2.840 (2.340, 3.460)	0.405
FOL (ng/mL), median (IQR)	9.250 (6.473, 12.205)	8.760 (6.190, 12.010)	10.940 (8.920, 15.930)	< 0.001
INS (mU/L), median (IQR)	7.200 (5.300, 10.700)	6.800 (5.200, 9.700)	9.900 (6.120, 16.150)	< 0.001
Serum GPNMB conc (pg/mL), median (IQR)	13522 (6175, 19496)	12389 (5351, 18039)	20047 (15242, 24870)	< 0.001
Cataract (%)	21.400	17.630	43.330	< 0.001

Data are expressed as median (interquartile range), or %. BMI body mass index, FMI fat mass index, Fat% body fat percentage, HOMA-IR the homeostasis model assessment of insulin resistance, HbA1c glycated hemoglobin, GLU fasting blood-glucose, TC total cholesterol, TG triglyceride, HDL-C HDL cholesterol, LDL-C LDL cholesterol, FOL folic acid, INS insulin.

P values for student’s t test or Wilcoxon rank sum test or Chi square test.

**Figure 3:**
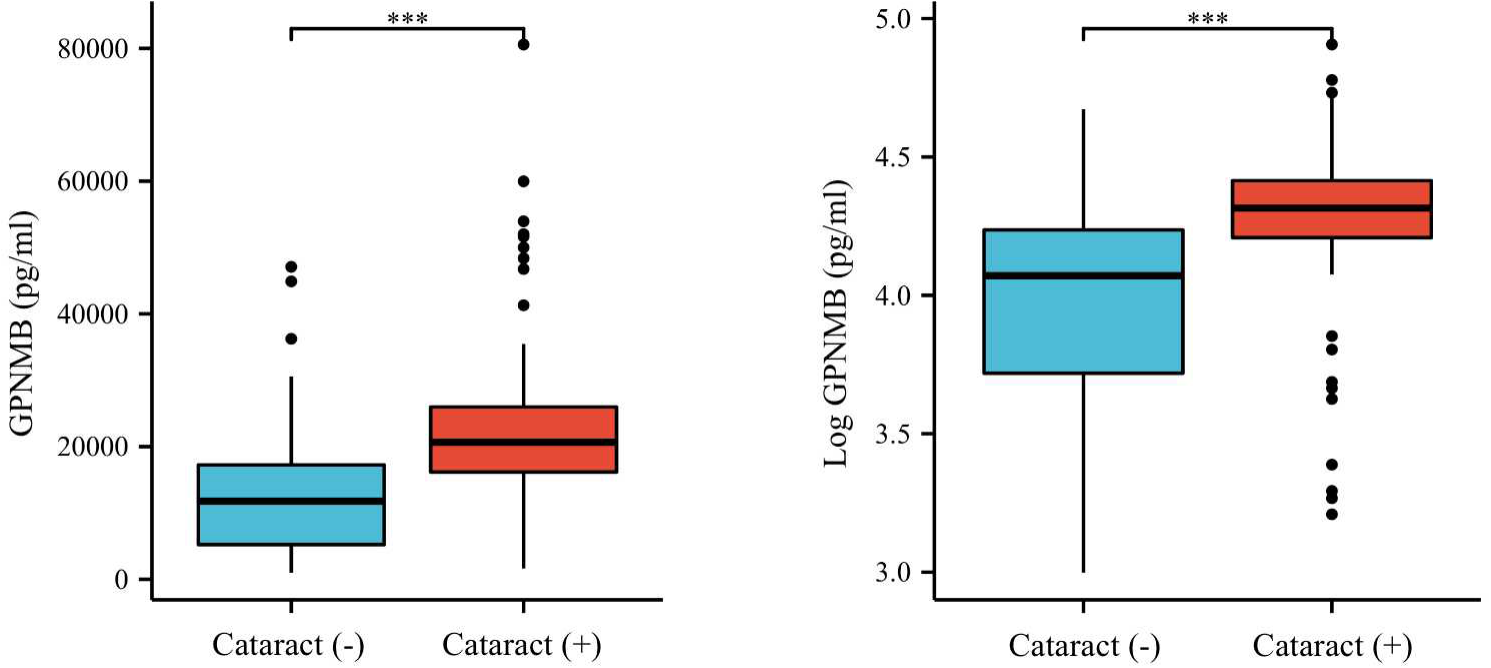
Plasma GPNMB levels depending on the existence of cataract. ***P < 0.001 using a Wilcoxon rank sum test.

**Figure 4:**
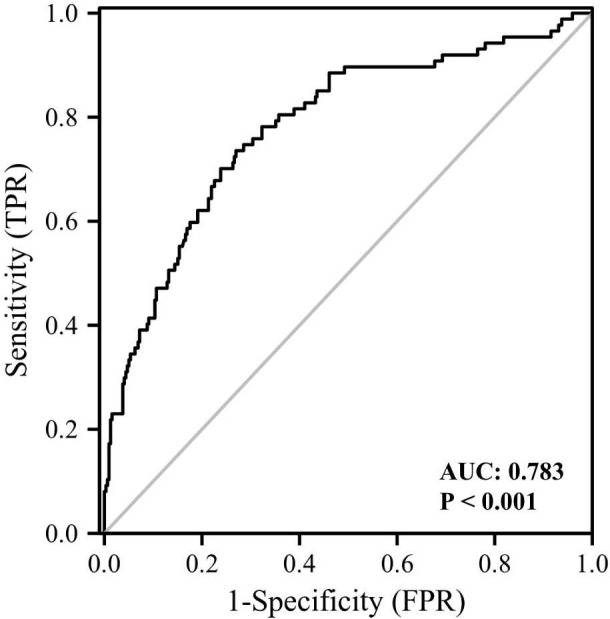
ROC curve analysis of the ability of plasma GPNMB to predict the presence of cataract. AUC, area under curve.

We then compared the variables in subjects with or without cataract (**Table 2**). Those with cataract were older and had higher FMI, Fat%, HOMA-IR, HbA1c, TC, TG, FOL, INS, blood glucose and serum GPNMB levels than those without cataract. A higher proportion of subjects with DM were detected in the cataract group than the cataract-free group. Furthermore, we performed analysis in subjects with DM and found that DM-bearing participants tended to be older and had a higher level of TC and serum GPNMB (**Table 3**). Circulating serum GPNMB levels were higher in subjects with cataract than those without cataract (**Figure 3**). The ability of serum GPNMB levels to predict the presence of cataract was 0.783, based on ROC curve analysis (asymptotic significance: <0.001) (**Figure 4**). The optimal cut-off value of GPNMB was 16,630.675 pg/mL (73.6% sensitivity and 73.0% specificity) to detect DM.

**Table 2:** Clinical and biochemical characteristics of study participants classified according to cataract.

Characteristic	Overall	Cataract (-), n=319	Cataract (+), n=87	*P* value
Age (years), median (IQR)	50.000 (34.000, 63.750)	45.000 (32.000, 55.000)	71.000 (65.000, 79.000)	< 0.001
Male (%)	38.200	39.810	32.180	0.241
BMI (kg/m^2^), median (IQR)	23.700 (21.600, 26.040)	23.500 (21.400, 25.800)	24.300 (22.530, 26.600)	0.064
FMI (kg/m^2^), median (IQR)	6.090 (5.038, 7.570)	5.940 (4.920, 7.360)	6.960 (5.880, 8.460)	0.001
Fat (%), mean ± SD	26.250 ± 8.2	26.120 ± 5.860	29.420 ± 5.730	< 0.001
HOMA-IR, median (IQR)	1.720 (1.200, 2.731)	1.620 (1.160, 2.460)	2.180 (1.460, 3.200)	< 0.001
HbA1c (%), median (IQR)	5.800 (5.600, 6.200)	5.800 (5.500, 6.100)	5.900 (5.700, 6.400)	0.001
GLU (mmol/l), median (IQR)	5.200 (5.000, 5.700)	5.200 (4.900, 5.600)	5.600 (5.200, 6.300)	< 0.001
TC (mmol/l), median (IQR)	4.740 (4.180, 5.363)	4.680 (4.180, 5.260)	4.970 (4.250, 5.620)	0.047
TG (mmol/l), median (IQR)	0.960 (0.640, 1.470)	0.890 (0.620, 1.400)	1.110 (0.840, 1.560)	0.002
HDL-C (mmol/l), median (IQR)	1.420 (1.190, 1.690)	1.420 (1.190, 1.680)	1.430 (1.210, 1.740)	0.451
LDL-C (mmol/l), median (IQR)	2.860 (2.335, 3.502)	2.830 (2.340, 3.390)	3.090 (2.310, 3.570)	0.252
FOL (ng/mL), median (IQR)	9.250 (6.473, 12.205)	8.580 (5.980, 11.660)	10.950 (8.540, 19.630)	< 0.001
INS (mU/L), median (IQR)	7.200 (5.300, 10.700)	6.800 (5.200, 10.250)	8.100 (5.900, 11.750)	0.011
Serum GPNMB conc. (pg/mL), median (IQR)	13522 (6175, 19496)	11778 (5230, 17232)	20658 (16163, 25958)	< 0.001
Diabetes (%)	14.800	10.660	29.890	< 0.001

Data are expressed as median (interquartile range), or %. BMI body mass index, FMI fat mass index, Fat% body fat percentage, HOMA-IR the homeostasis model assessment of insulin resistance, HbA1c glycated hemoglobin, GLU fasting blood-glucose, TC total cholesterol, TG triglyceride, HDL-C HDL cholesterol, LDL-C LDL cholesterol, FOL folic acid, INS insulin.

P values for student’s t test or Wilcoxon rank sum test or Chi square test.

**Table 3:** Clinical and biochemical characteristics of study participants with diabetes classified according to cataract.

Characteristic	Overall	Cataract (-), n=34	Cataract (+), n=26	*P* value
Age (years), median (IQR)	66.017 (53.942, 78.092)	60.235 (48.754, 71.716)	73.577 (65.502, 81.653)	< 0.001
Male (%)	45.000	21.700	23.300	0.228
BMI (kg/m^2^), median (IQR)	24.690 (23.000, 27.230)	24.450 (21.750, 26.945)	24.690 (23.900, 27.400)	0.298
FMI (kg/m^2^), median (IQR)	7.170 (5.915, 8.045)	6.870 (5.743, 8.028)	7.460 (6.440, 8.230)	0.346
Fat (%), mean ± SD	29.360 ± 5.820	29.032 ± 6.217	29.973 ± 5.144	0.619
HOMA-IR, median (IQR)	3.046 (1.787, 4.465)	2.555 (1.446, 4.415)	3.280 (1.870, 4.480)	0.486
HbA1c (%), median (IQR)	6.574 (5.528, 7.620)	6.485 (5.425, 7.545)	6.655 (5.603, 7.706)	0.606
GLU (mmol/l), median (IQR)	6.300 (5.575, 7.075)	6.000 (5.650, 6.800)	6.400 (5.200, 7.500)	0.783
TC (mmol/l), median (IQR)	4.583 (3.490, 5.678)	4.940 (4.200, 5.605)	4.510 (3.610, 4.710)	0.043
TG (mmol/l), median (IQR)	1.160 (0.888, 1.630)	1.220 (0.915, 1.780)	1.110 (0.890, 1.340)	0.323
HDL-C (mmol/l), median (IQR)	1.225 (1.058, 1.435)	1.320 (1.115, 1.480)	1.150 (1.050, 1.410)	0.447
LDL-C (mmol/l), median (IQR)	2.825 (1.912, 3.738)	3.051 (2.224, 3.878)	2.572 (1.618, 3.525)	0.055
FOL (ng/mL), median (IQR)	10.940 (8.920, 15.930)	10.410 (8.720, 12.122)	12.200 (9.530, 19.640)	0.154
INS (mU/L), median (IQR)	9.900 (6.125, 16.150)	9.550 (6.525, 15.875)	10.900 (6.125, 16.150)	0.707
Serum GPNMB conc. (pg/mL), median (IQR)	20050 (15240, 24870)	17270 (14110, 23060)	22710 (18870, 31080)	0.013

Data are expressed as median (interquartile range), or %. BMI body mass index, FMI fat mass index, Fat% body fat percentage, HOMA-IR the homeostasis model assessment of insulin resistance, HbA1c glycated hemoglobin, GLU fasting blood-glucose, TC total cholesterol, TG triglyceride, HDL-C HDL cholesterol, LDL-C LDL cholesterol, FOL folic acid, INS insulin.

P values for student’s t test or Wilcoxon rank sum test or Chi square test.

**Table 4** shows the general characteristics of enrolled individuals grouped according to GPNMB tertiles. Lower age, Fat%, TC, TG, LDL-C, FOL levels, and more men were detected in tertile 1 than in tertiles 2 and 3. Nevertheless, the median levels of TC and LDL-C of individuals in tertile 2 were higher than those in tertile 3. Notably, a higher proportion of participants in tertile 3 were diagnosed as having DM and cataract than in tertiles 2 and 1.

**Table 4:** General characteristics of patients by tertiles of plasma GPNMB level.

Characteristic	Tertiles of circulating GPNMB levels			*P* value
	Tertile 1 (<9650 pg/mL) n = 135	Tertile 2 (9650-17460 pg/mL) n = 135	Tertile 3 (>17460 pg/mL) n = 136	
Age (years), median (IQR)	42.000 (31.000, 53.500)	48.000 (34.000, 58.500)	63.500 (49.750, 73.500)	< 0.001
Male (%)	46.670	34.070	33.820	0.046
BMI (kg/m^2^), median (IQR)	23.900 (21.500, 25.950)	23.450 (21.680, 25.660)	23.550 (21.670, 26.320)	0.860
FMI (kg/m^2^), median (IQR)	5.900 (4.840, 7.360)	6.160 (5.170, 7.400)	6.540 (5.170, 7.890)	0.274
Fat%, mean ± SD	25.510 ± 6.050	27.040 ± 5.970	27.540 ± 5.640	0.022
HOMA-IR, median (IQR)	1.630 (1.210, 2.430)	1.720 (1.150, 2.430)	1.840 (1.200, 3.090)	0.274
HbA1c%, median (IQR)	5.800 (5.570, 6.100)	5.800 (5.600, 6.100)	5.900 (5.600, 6.300)	0.297
GLU (mmol/l), median (IQR)	5.200 (5.000, 5.600)	5.200 (4.900, 5.700)	5.300 (5.000, 6.000)	0.085
TC (mmol/l), median (IQR)	4.620 (4.170, 5.150)	4.900 (4.260, 5.500)	4.680 (4.170, 5.300)	0.047
TG (mmol/l), median (IQR)	0.850 (0.600, 1.340)	0.930 (0.640, 1.390)	1.040 (0.750, 1.520)	0.040
HDL-C (mmol/l), median (IQR)	1.380 (1.160, 1.600)	1.450 (1.230, 1.710)	1.420 (1.170, 1.710)	0.407
LDL-C (mmol/l), median (IQR)	2.760 (2.220, 3.340)	3.080 (2.500, 3.580)	2.820 (2.340, 3.460)	0.045
FOL (ng/mL), median (IQR)	7.940 (5.450, 10.950)	9.530 (6.870, 12.290)	10.300 (7.190, 15.320)	< 0.001
INS (mU/L), median (IQR)	6.950 (5.470, 10.100)	7.000 (5.350, 9.550)	7.550 (5.230, 11.750)	0.437
Cataract (%)	6.670	13.330	44.120	< 0.001
Diabetes (%)	4.440	12.590	27.210	< 0.001

Data are expressed as median (interquartile range), or %. BMI body mass index, FMI fat mass index, Fat% body fat percentage, HOMA-IR the homeostasis model assessment of insulin resistance, HbA1c glycated hemoglobin, GLU fasting blood-glucose, TC total cholesterol, TG triglyceride, HDL-C HDL cholesterol, LDL-C LDL cholesterol, FOL folic acid, INS insulin.

P values for ANOVA or Kruskal-Wallis H test or Chi square test.

As shown in **Table 5**, univariable logistic regression analysis revealed that age, BMI, FMI, Fat%, HOMA-IR, HbA1c, GLU, INS, TC, HDL-C, FOL, and log GPNMB were associated with DM. We included these variables in the multivariable logistic regression analysis. To avoid collinearity, we explored the linear correlation of log GPNMB with age, Fat%, GLU, TC, and FOL through Pearson’s correlation coefficient analysis (**Supplementary Figure 1**) and finally excluded those five variables from the multivariable logistic regression analysis. It was indicated that HOMA-IR, HDL-C, and log GPNMB were independently associated with the presence of DM.

**Table 5:** Univariable and multivariable logistic regression analyses for diabetes.

	Association with presence of diabetes			
	Single		Multiple	
	OR (95% CI)	P value	OR (95% CI)	P value
Age	1.076 (1.054-1.098)	<0.001	–	–
Gender	1.393 (0.801-2.424)	0.240	–	–
BMI	1.120 (1.031-1.216)	0.007	0.954 (0.743-1.225)	0.712
FMI	1.259 (1.102-1.438)	0.001	1.040 (0.739-1.464)	0.820
Fat (%)	1.092 (1.034-1.154)	0.002	–	–
HOMA-IR	1.322 (1.143-1.530)	<0.001	3.363 (1.101-10.268)	0.033
HbA1c	3.312 (1.966-5.581)	<0.001	1.207 (0.556-2.619)	0.634
GLU	2.484 (1.812-3.405)	<0.001	–	–
INS	1.052 (1.016-1.090)	0.005	0.753 (0.557-1.107)	0.064
TC	0.719 (0.518-0.998)	0.049	–	–
TG	1.195 (0.860-1.661)	0.289	–	–
HDL-C	0.186 (0.069-0.499)	0.001	0.171 (0.033-0.889)	0.036
LDL-C	0.805 (0.566-1.145)	0.227	–	–
FOL	1.082 (1.034-1.132)	0.001	–	–
Log GPNMB	14.871 (4.867-45.439)	<0.001	6.626 (1.316-33.370)	0.022

The symbol - indicates the cells without data.

Data are expressed as median (interquartile range), or %. BMI body mass index, FMI fat mass index, Fat% body fat percentage, HOMA-IR the homeostasis model assessment of insulin resistance, HbA1c glycated hemoglobin, GLU fasting blood-glucose, TC total cholesterol, TG triglyceride, HDL-C HDL cholesterol, LDL-C LDL cholesterol, FOL folic acid, INS insulin.

Log-transformation was used for GPNMB before statistical analysis. P values for univariable or multivariable logistic regression analysis.

**Figure 5:**
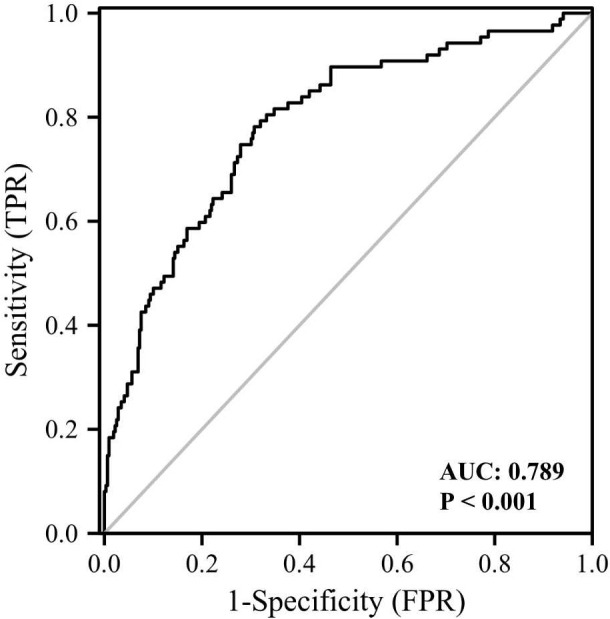
ROC curve analysis of the ability of plasma GPNMB and the presence of diabetes together to predict the presence of cataract. AUC, area under curve.

We also performed univariable and multivariable logistic regression analysis to investigate potential biomarkers for cataract (**Table 6**). Age, FMI, Fat%, HbA1c, GLU, TC, FOL, and log GPNMB were found to be associated with cataract by univariable logistic regression analysis. Multivariable logistic regression analysis showed that log GPNMB was an independent predictor of cataract. To gain further understanding of the relationship between serum GPNMB and DM-related cataract, we utilized ROC curve analysis based on serum GPNMB levels and the diabetic status (**Figure 5**). The area under the ROC curve was 0.789 with an asymptotic significance <0.001.

**Table 6:** Univariable and multivariable logistic regression analyses for cataract.

	Association with presence of Cataract			
	Single		Multiple	
	OR (95% CI)	*P* value	OR (95% CI)	*P* value
Age	1.209 (1.158-1.261)	<0.001	–	–
Gender	0.717 (0.434-1.186)	0.195	–	–
BMI	1.040 (0.966-1.119)	0.299	–	–
FMI	1.190 (1.053-1.345)	0.005	1.074 (0.920-1.253)	0.364
Fat (%)	1.080 (1.000-1.167)	<0.001	–	–
HOMA-IR	1.104 (0.978-1.245)	0.109	–	–
HbA1c	1.643 (1.115-2.421)	0.012	1.569 (1.000-2.463)	0.050
GLU	1.495 (1.196-1.869)	<0.001	–	–
INS	1.019 (0.995-1.042)	0.117	–	–
TC	1.336 (1.040-1.716)	0.023	–	–
TG	1.189 (0.897-1.576)	0.229	–	–
HDL-C	1.488 (0.754-2.933)	0.252	–	–
LDL-C	1.215 (0.921-1.604)	0.169	–	–
FOL	1.142 (1.095-1.192)	<0.001	–	–
Log GPNMB	36.785 (11.908-113.634)	<0.001	9.867 (2.837-34.324)	<0.001

The symbol - indicates the cells without data.

Data are expressed as median (interquartile range), or %. BMI body mass index, FMI fat mass index, Fat% body fat percentage, HOMA-IR the homeostasis model assessment of insulin resistance, HbA1c glycated hemoglobin, GLU fasting blood-glucose, TC total cholesterol, TG triglyceride, HDL-C HDL cholesterol, LDL-C LDL cholesterol, FOL folic acid, INS insulin.

Log-transformation was used for GPNMB before statistical analysis. P values for univariable or multivariable logistic regression analysis.

## Discussion

To our best knowledge, our study is the first to demonstrate that serum GPNMB levels correlate with both DM and cataract. The results showed higher GPNMB levels in subjects with DM or cataract than in control subjects. In addition, serum GPNMB levels were independently correlated with both DM and cataract. ROC curve analysis also proved the potential of serum GPNMB levels as an independent biomarker for DM and cataract. Notably, the ability of GPNMB levels to diagnose cataract was improved with the presence of DM. Further research is required to validate these results.

GPNMB possesses an extracellular N-terminal signal peptide, a transmembrane domain, and a short cytoplasmic tail (21) and functions both as an inflammatory mediator and a circulating cytokine. Referring to its role in inflammatory response, a previous study detected the prevailing expression of GPNMB in macrophages, and showed that the expression level of GPNMB can be induced by interferon gamma (IFN-γ) and lipopolysaccharide and is elevated in inflammatory macrophages (15). In addition, overexpression of GPNMB in RAW264.7 cells, a macrophage cell line derived from mice, causes significant decrease of interleukin (IL)-6 and IL-12p40, inflammatory cytokines, as well as the inflammatory regulator nitric oxide (NO) (15). Thus, GPNMB is considered as a negative regulator of macrophage inflammatory capacity. Furthermore, macrophage inflammatory response is closely related to metabolic disorders (29). and DM is one of the most common metabolic diseases and can cause aberrant metabolism of blood glucose and induce the inflammatory response. By utilizing mouse models fed with high-fat diet, Prabata et al. (30) detected attenuated insulin and glucose tolerance in GPNMB-knock-out (KO) mice, and observed high levels of inflammatory cytokines produced by macrophages derived from GPNMB KO mice. In addition, the increase in inflammatory cytokines secreted by macrophages could be abrogated by added GPNMB extracellular domain. Prabata et al. (30) also found that GPNMB could bind to CD44 to prohibit nuclear factor kappa-B (NF-κB), thus abate the inflammatory response of macrophages.

GPNMB can also be cleaved into a soluble form that contains an ectodomain (ECD) and functions as a secreted cytokine (31). Gong et al. (20) revealed that GPNMB-ECD could interact with CD44 to trigger AKT signaling and further contributed to lipogenesis in adipocytes of withe adipose tissues. They further showed that GPNMB was capable of inducing obesity and insulin resistance in mice and this phenotype could be rescued by an anti-GPNMB antibody. Gong et al. (20) also performed a population-based study and ascertained that serum levels of GPNMB were correlated with human obesity. Research also determined the positive association between serum levels of GPNMB and disease presence and severity of Parkinson’s Disease (32). Another study that concentrated on diabetic retinopathy—a microvascular complication characterized by aberrant angiogenesis—found that GPNMB knockdown attenuated retinal angiogenesis stimulated by high glucose both *in vivo* and *in vitro (* 33).

In this study, elevated serum levels of GPNMB were determined in subjects with DM and cataract. Based on the results of previous research, it was quite possible that the increased circulating levels of GPNMB caused abnormal phosphorylation of AKT *via* binding to CD44 and then stimulated anomalous activation of AKT/PI3K signaling to promote lipogenesis and provoke diabetes. Moreover, activation of AKT signaling followed by downregulation of connexin 43 is required for transforming growth factor-beta 2 (TGF-β2)-induced epithelial mesenchymal transition (EMT) of HLE B-3 cells—a human lens epithelial cell line (34). It has been reported that lens epithelial cells that undergo EMT can result in posterior capsule opacification, which is the main cause and symptom of cataract (35). Yao et al. (36) demonstrated that integrin beta-1, a target protein of GPNMB, was essential for TGF-β2-mediated migration of lens epithelial cells. Thus, the raised serum levels of GPNMB may abate the expression of connexin 43 and stimulate the upregulation of integrin beta-1 to promote cataract formation. Notably, high glucose levels due to diabetes-induced insulin resistance also contributes to the pathogenesis of cataract (37). Accordingly, we concluded that the way in which GPNMB promoted DM might enhance the development of cataract. Further investigations are needed to reveal the underlying molecular mechanisms.

To minimize probable bias, we excluded subjects with alcohol and drug abuse, severe diseases, and those that underwent recent medical treatment. We aimed to only enroll subjects with a relatively steady metabolism, because DM and cataract are both metabolic diseases. Moreover, recruited individuals were all required to undergo 8h fasting before screening to maintain stable levels of serum cytokines. We reviewed the medical history of included individuals and performed ocular examination to avoid excluding those who had undergone cataract surgery before or were unaware of their cataract. Furthermore, we utilized different statistical methods to analyze the data, and found that the results were consistent. These strategies added to the strengths of our study. A limitation of the study is the relatively small number of individuals in the subgroups. Further mechanistic studies are required to investigate the mechanism underlying GPNMB’s effect on the pathogenesis of DM-related cataract.

## Data availability statement

The raw data supporting the conclusions of this article will be made available by the authors, without undue reservation.

## Ethics statement

The studies involving human participants were reviewed and approved by the Research Ethics Committee of Beijing Hospital (2019BJYYEC-054-02). The patients/participants provided their written informed consent to participate in this study.

## Author contributions

Conceptualization: JC, J-PC, T-MZ. Methodology: JC, DH, Y-YL. Samples collection: DH, Y-YL, C-BL, L-TZ, G-QF, YW, L-QZ, JP, C-BL. Software Formal analysis: DH, Y-YL, CZ, X-FL. Data curation: JC, DH, Y-YL. Writing—original draft preparation: DH, Y-YL. Writing—review and editing: JC, J-PC, T-MZ, JP, L-QZ, TS. Supervision: JC, J-PC. All authors contributed to the article and approved the submitted version.

## Supplementary material

Supplementary Figure 1 The linear correlation of log GPNMB with different variables through Pearson’s correlation coefficient analysis. *BMI* body mass index, *FMI* fat mass index, *Fat%* body fat percentage, *HOMA-IR* the homeostasis model assessment of insulin resistance, *HbA1c* glycated hemoglobin, *GLU* fasting blood-glucose, *TC* total cholesterol, *HDL-C* HDL cholesterol, *FOL* folic acid, *INS* insulin.
