# Supplementary figures and images for: Serum glycoprotein non-metastatic melanoma protein B (GPNMB) level as a potential biomarker for diabetes mellitus-related cataract: A cross-sectional study
Source: Front Endocrinol (Lausanne). 2023 Feb 16;14:1110337. doi: 10.3389/fendo.2023.1110337 (PMC9978497; doi:10.3389/fendo.2023.1110337)

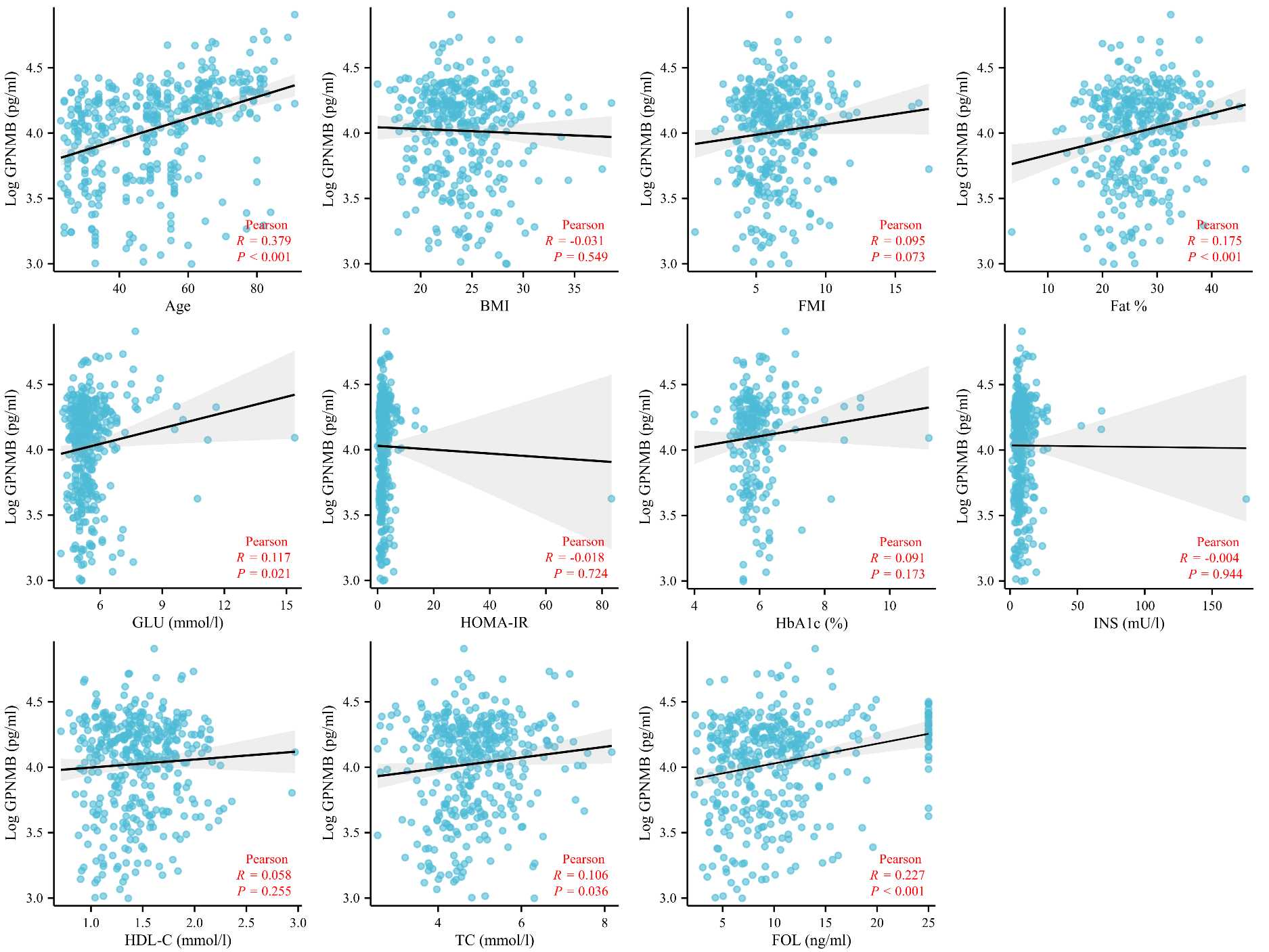

Supplement: Supplementary Figure 1 — The linear correlation of log GPNMB with different variables through Pearson’s correlation coefficient analysis. BMI body mass index, FMI fat mass index, Fat% body fat percentage, HOMA-IR the homeostasis model assessment of insulin resistance, HbA1c glycated hemoglobin, GLU fasting blood-glucose, TC total cholesterol, HDL-C HDL cholesterol, FOL folic acid, INS insulin. [file Image_1.jpg]
